# Prevalence and Genetic Characterization of *mcr-1*-Positive *Escherichia coli* Isolated from Retail Meats in South Korea

**DOI:** 10.4014/jmb.2007.07008

**Published:** 2020-09-21

**Authors:** Seokhwan Kim, Hansol Kim, Hai-Seong Kang, Yonghoon Kim, Migyeong Kim, Hyosun Kwak,, Sangryeol Ryu

**Affiliations:** 1 Division of Food Microbiology, National Institute of Food and Drug Safety Evaluation, Cheongju 2859, Republic of Korea; 2 Department of Food and Animal Biotechnology, Department of Agricultural Biotechnology, Seoul National University, Seoul 0886, Republic of Korea

**Keywords:** Colistin resistance, *mcr-1*, prevalence, IncI2 plasmid, IncX4 plasmid, retail meats

## Abstract

The spread of plasmid-mediated colistin resistance has posed a serious threat to public health owing to its effects on the emergence of pandrug-resistant bacteria. In this study, we investigated the prevalence and characteristics of *mcr-1*-positive *Escherichia coli* isolated from retail meat samples in Korea. In total, 1,205 *E. coli* strains were isolated from 3,234 retail meat samples in Korea. All *E. coli* strains were subjected to antimicrobial susceptibility testing and were examined for the presence of *mcr-1* gene. All *mcr-1*-positive *E. coli* (*n* = 10, 0.8%) from retail meat were subjected to pulse-field gel electrophoresis (PFGE) and whole-genome sequencing (WGS). The transferability of *mcr-1* gene was determined by conjugation assays. The *mcr-1*-positive strains exhibited diverse clonal types. Our *mcr-1* genes were located in plasmids belonged to the IncI2 (*n* = 1) and IncX4 (*n* = 8) types, which were reported to be prevalent in Asia and worldwide, respectively. Most *mcr-1* genes from *mcr-1*-positive strains (9/10) were transferable to the recipient strain and the transfer frequencies ranged from 2.4 × 10^-3^ to 9.8 × 10^-6^. Our data suggest that the specific types of plasmid may play an important role in spreading plasmid-mediated colistin resistance in Korea. Furthermore, our findings suggest that the retail meat may be an important tool for disseminating plasmid-mediated colistin resistance.

## Introduction

Colistin is a cationic polypeptide antibiotic that acts against most gram-negative bacteria, including those from the family *Enterobacteriaceae*. Colistin is a type of polymyxin, the five known subtypes of which (A–E) lead to disruption of membrane permeability by mediating electrostatic interactions between positively charged residues of polymyxin and negatively charged lipid A components of lipopolysaccharides (LPSs) in the bacterial membrane [1]. The use of colistin, also known as polymyxin E, in clinical practice has been limited because of its nephrotoxicity and neurotoxicity [2, 3]. However, owing to recent increases in multidrug resistant gram-negative bacteria and the rapid expansion of carbapenemase-producing *Enterobacteriaceae*, colistin has re-emerged as the last treatment option for severe bacterial infections [4, 5].

Several studies have reported that colistin resistance is related to chromosomal mutations in two-component systems, such as PmrAB and PhoPQ, leading to modification of LPS moieties in the outer membrane [6, 7]; therefore, there was little concern regarding the spread of colistin resistance. However, since Liu and colleagues first described the plasmid-mediated transfer of colistin resistance in China in 2015 [8], the spread of the mobile colistin resistance gene has posed a serious threat to human health because of the possible emergence of bacteria resistant to all available antimicrobials [9, 10].

The mobile colistin resistance gene *mcr-1* encodes a phosphoethanolamine transferase enzyme that is capable of modifying lipid A in the bacterial membrane and reducing the affinity for colistin [11]. Most *mcr-1* genes are located on various types of plasmids, including IncI2-, IncX4-, IncHI2-, IncP-, IncY-, and IncFI1-type plasmids, and are easily transferred to other strains [12, 13]. These *mcr-1* genes were identified globally in various species of *Enterobacteriaceae*, such as *Escherichia coli* and *Salmonella enterica* isolated from humans, food animals, foods, and the environment [14]. *mcr-1*-positive *E. coli* have also been found in humans and food animals in Korea [15-17]. However, few studies have reported *mcr-1*-bearing bacteria recovered from food samples in Korea [18].

Since 2003, the Korean government has monitored and surveyed the antimicrobial resistance of bacteria collected from foods, such as retail meats, within the framework of the National Program on Antimicrobial Resistance Management [19]. *mcr-1*-positive *E. coli* were recently identified from retail meat samples. In this study, we report the occurrence rates of *mcr-1*-positive *E. coli* from retail raw meats in Korea and their genetic characteristics.

## Materials and Methods

### Sample Collection and Bacterial Isolation

In total, 3,234 raw meat samples, including beef (*n* = 1,290), pork (*n* = 1,126), and chicken (*n* = 818), were purchased at 291 retail stores spread across all the provinces of South Korea between 2015 and 2018. Overall, an average of ~800 raw meat samples were purchased per year. The domestic meat samples were obtained from 43 reputable processing companies for beef, 32 for pork, and 18 for chicken. Among the imported meat samples, beef samples were from 5 countries, pork from 14 countries, and chicken from 4 countries (Table S1). The meat samples were kept on ice during transportation from the grocery stores to the laboratory. Twenty-five grams of each meat sample was homogenized with 225 ml EC broth (Difco, USA) using a stomacher. The EC broth was incubated under aerobic conditions at 37°C for 24 h. An aliquot of each sample was streaked onto selective medium of Eosin Methylene Blue agar (Oxoid, UK) and incubated at 37°C for 24 h. Typical *E. coli* colonies (green metallic sheen) were sub-cultured on nutrient agar (Difco) and confirmed using a Vitek 2 Compact microbial identification system (bioMérieux, France) or Vitek MS (bioMérieux) in accordance with the manufacturer’s instructions. One typical and well-isolated *E. coli* strain per meat sample was selected. If no typical growth was observed, the sample was treated as a negative sample and was discarded. All isolates were stored at -80°C in Tryptic Soy Broth (Difco), mixed with 15% glycerol.

### Antimicrobial Susceptibility

All *E. coli* strains (*n* = 1,205) were subjected to antimicrobial susceptibility testing using the following antibiotics: amoxicillin/clavulanic acid (AmC), ampicillin (AMP), cefoxitin (FOX), ceftiofur (CTF), chloramphenicol (CHL), ciprofloxacin (CIP), colistin (COL), gentamicin (GEN), nalidixic acid (NAL), streptomycin (STR), tetracycline (TET), and trimethoprim/sulfamethoxazole (SXT). The minimum inhibitory concentrations (MICs) of these antimicrobials were determined by a broth-microdilution method using a commercially available Sensititre plate KRNV4F (Trek Diagnostic Systems, USA). *E. coli* ATCC 25922 was used as a reference strain. For screening of colistin-resistant *E. coli*, the breakpoint for colistin resistance was applied at greater than 2 μg/ml, by referring to the European Committee on Antimicrobial Susceptibility Testing guidelines [20]. Susceptibility results of MICs for other antibiotics were interpreted in accordance with the Clinical and Laboratory Standards Institute guidelines [21] and the National Antimicrobial Resistance Monitoring System [22].

### Polymerase Chain Reaction (PCR) Amplification of *mcr-1* Genes

Template DNA from *E. coli* isolates for PCR was prepared using an UltraClean Microbial DNA Isolation Kit (MO BIO Laboratories Inc., USA) following the manufacturer’s instructions and stored at -20°C until use. The presence of *mcr-1* was detected by PCR amplification using previously described primers [8]. A DNA thermal cycler (C1000 PCR System; Bio-Rad, USA) was used for PCR amplification, with the following protocol: 94°C for 15 min; 25 cycles of 94°C for 30 sec, 58°C for 90 sec, and 72°C for 60 sec; and final extension at 72°C for 10 min. The PCR products were analyzed using 1.5% (w/v) agarose gel electrophoresis.

### Conjugation Assay of *mcr-1*-Positive Isolates

The transmissibility of *mcr-1* gene was determined by conjugation assays in accordance with previously described broth-mating methods [23]. Briefly, azide-resistant *E. coli* strain J53 and isolates bearing mcr genes were used as the recipient and donor, respectively. Recipient and donor strains were mixed and incubated at a ratio of 1:1 in Luria-Bertani broth (Difco) for 8 h. Aliquots of these mixtures were plated on tryptic soy agar (Difco) containing sodium azide (200 μg/ml) and colistin (4 μg/ml) and incubated at 37°C for 20 h. PCR was used to confirm that the transconjugants carried the *mcr-1* gene. Conjugation frequencies were determined as the number of transconjugants per a donor cell.

### Pulsed-Field Gel Electrophoresis (PFGE) of *mcr-1*-Positive Isolates

Genotyping of *mcr-1*-positive *E. coli* isolates was conducted by PFGE with the CHEF-Mapper system (Bio-Rad) according to the PulseNet standardized protocol (http://www.pulsenetinternational.org/protocols/). Genomic DNA was digested with *Xba*I (Roche Molecular Biochemicals, USA) and separated on 1.0% pulsed-field certified agarose. Running conditions were as follows: 6.0 V/cm at 14°C for 18 h, with pulse times ramped from 2.2 to 54.2 s in 0.5× Tris-borate-ethylenediaminetetraacetic acid buffer. Genomic DNA from *Salmonella enterica* Braenderup H9812 (ATCC BAA-664) restricted with *Xba*I was used as a size marker. The PFGE patterns were analyzed with BioNumerics software ver. 5. 1 (Applied Maths, Belgium) using the Dice similarity coefficient with a 1.5% position tolerance, and clustering was performed by the unweighted-pair group method with average linkages (UPGMA). The results were interpreted according to the criteria reported previously [24].

### Whole-Genome Sequencing (WGS) of *mcr-1*-Positive Isolates

WGS and assembly were performed at ChunLab Inc. (Korea) and Senigen Inc. (Korea). High-quality genomic DNA was extracted using an UltraClean Microbial DNA Isolation Kit (MO BIO Laboratories Inc.) according to the manufacturer’s instructions. The whole genome of *mcr-1*-positive isolates was sequenced on an Illumina Miseq desktop sequencer (Illumina Inc., USA), with paired-end reads of 300 bp length. The sequencing library was prepared with a TruSeq DNA LT Sample Prep Kit (Illumina Inc.) for the Illumina system. A de novo assembly was performed using SPAdes genome assembler version 3.13.0 [25]. The number of assembled contigs ranged between 69 and 135, with an average sequencing coverage of 129×. Antibiotic-resistance genes, replicon typing, and multilocus sequence typing (MLST) were conducted in ResFinder 3.1, PlasmidFinder 2.0, and MLST 2.0, respectively, on the Center for Genomic Epidemiology website (http://www.genomicepidemiology.org) [26-28]. Genome sequences were compared using the BLAST Ring Image Generator (BRIG) [29].

The whole-genome sequencing data reported in this study have been deposited at GenBank with the following accession numbers: from JACABR000000000 to JACABY000000000, WVVJ00000000, and WVVM00000000 (Table S2).

### Statistical Data Analysis

Statistical analysis was performed using Epitools [30]. Comparisons between groups were evaluated by Chi-square (χ^2^) test. Results with *p* values of less than 0.05 were considered significant.

## Results

### Prevalence of *mcr-1*-Postive *E. coli* from Retail Meat

A total of 1,205 *E. coli* strains were isolated from 3,234 retail meat samples in Korea between 2015 and 2018. The colistin resistance of *E. coli* isolated from retail meat and the *mcr-1* carriage rates are shown in Table 1. Of the 1,205 *E. coli* isolates, 51 isolates (4.2%) were resistant to colistin. The colistin-resistant isolates were obtained from domestic meat (3.7%, 33/891) and imported meat (5.7%, 18/314). Among these isolates, the *mcr-1* gene was identified in 10 *E. coli* isolates, including one isolate from domestic pork, one from German pork, and eight from Brazilian chicken meat. However, 41 other-colistin-resistant isolates did not have the *mcr-1* gene. The prevalence rates of *mcr-1*-positive isolates from domestic and imported meats were 0.1% and 2.9%, respectively. Isolates from imported meat samples showed significantly higher *mcr-1* carriage rates than isolates from domestic meat samples (*p* < 0.05).

### Antimicrobial Resistance of Colistin-Resistant *E. coli*

Antimicrobial resistance of 51 colistin-resistant *E. coli* to 12 antibiotics is shown in Table 2. Among colistin-resistant strains, 34 strains (66.7%) exhibited a multidrug resistance (MDR) phenotype, which means that the strains were resistant to three or more antibiotics belonging to different categories. The occurrence of MDR in *mcr-1*-positive strains was not significantly different from that in *mcr-1*-negative strains. Moreover, no significant difference in the occurrence of resistance to each antimicrobial agent was present between *mcr-1*-positive strains and *mcr-1*-negative strains.

### Characteristics of *mcr-1*-Positive *E. coli*

Among *mcr-1*-positive strains, eight strains exhibited a resistance phenotypes to at least two and up to 10 (Table 3). In particular, two strains from domestic pork (EC2018_100) and Brazilian chicken meat (EC2017_I306) showed the extended-spectrum β-lactamase (ESBL) phenotype, which were previously reported from our group [31]. These two strains carried the *bla*_CTX-M-55_ gene and the *bla*_CTX-M-15_ gene, respectively. Meanwhile, four strains from imported meat samples harbored β-lactamase-related genes (*bla*_TEM_, *bla*_SHV_), which showed a non-ESBL phenotype and resistance to AMP. Three strains (EC2016_I15, EC2016_I115, and EC2018_100) exhibited the resistance to NAL or CIP. Although two of these strains (EC2016_I15 and EC2016_I115) did not harbor the quinolone resistance genes, the presence of point mutations in quinolone resistance-determining regions (QRDR) in chromosomal gyrA or parC genes was noted (Table S3). Resistance phenotypic results correlated with the presence of the different resistance genes for each antimicrobial family (Tables 3 and S3). All *mcr-1*-positive strains harbored the *mdf(A)* gene, but the resistance to macrolides was not determined in this study.

*mcr-1* genes, except EC2016_I182 strain, were found in the same contigs as replicons of the families of IncX4 (*n* =8) and IncI2 (*n* = 1), which means that *mcr-1* genes were located in the IncX4 and IncI2 plasmid. Additionally, *mcr-1*-positive strains contained a wide variety of plasmid incompatibility group replicons, ranging from one to seven per strain.

**Fig. 1:**
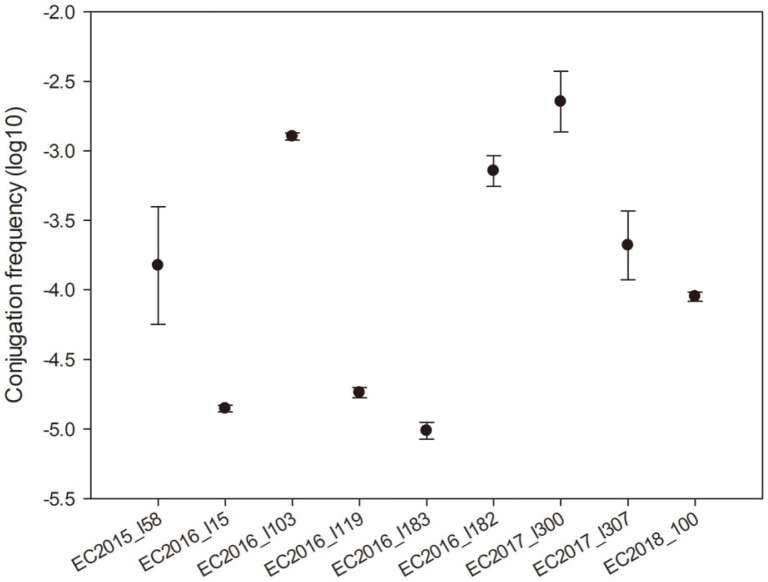
Conjugation frequencies of nine *mcr-1*-positive *E. coli* from retail meat. The data represent the averages and standard deviations.

Conjugation tests showed that 9 of the 10 *mcr-1*-positive strains were able to transfer their colistin-resistance phenotype to *E. coli* J53 (Table 3). Conversely, no other resistance among these strains was cotransferred to the recipient strain except the EC2018_100 strain. The transfer frequencies ranged from 2.4 × 10^-3^ to 9.8 × 10^-6^ (Fig. 1).

### Epidemiology of *mcr-1*-Positive *E. coli*

**Fig. 2:**
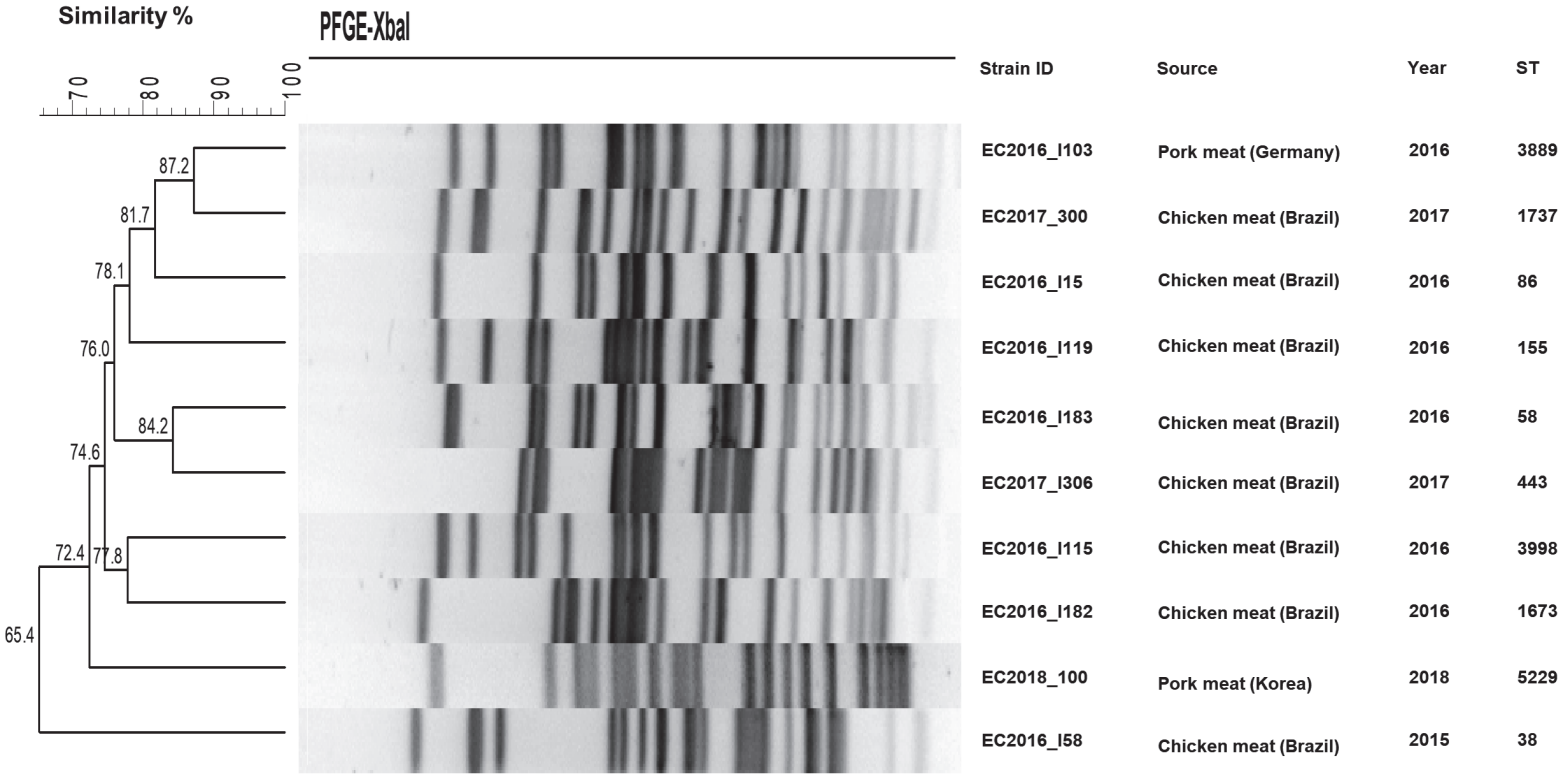
XbaI PFGE dendrogram with the corresponding MLST sequence types of *mcr-1*-positive *E. coli* strains from retail meat samples. Based on the UPGMA algorithm, the dendrogram revealed each different PFGE pulsotype.

In silico MLSTs of *mcr-1*-positive strains were generated from draft genome sequences, and sequence types (STs) were assigned according to the *E. coli* Achtman scheme. MLST indicated that nine strains belonged to different STs (Fig. 2). The ST (ST58) of one strain from Brazilian chicken meat was the clonal MLST complex of ST155. Additionally, all *mcr-1*-positive *E. coli* strains showed very diverse PFGE pulsotypes.

## Discussion

In our study, we detected the plasmid-mediated colistin resistance gene *mcr-1* in 10 *E. coli* strains from retail meat samples purchased at Korean grocery stores between 2015 and 2018. Amongst 10 *mcr-1*-positive *E. coli* strains, nine *E. coli* strains were recovered from the imported meat samples, and one *E. coli* strain was isolated from a domestic pork sample in 2018. The observed 0.1% prevalence of *mcr-1*-positive strains from retail domestic meat samples is comparable with the previously reported 0.1% prevalence in livestock in Korea [16]. Moreover, the prevalence of *mcr-1*-positive strains from the domestic meat in our study was lower than that of isolates previously reported from other countries, such as Brazil, China, Germany, Japan, the Netherlands, and Portugal [8,32-36]. Further, the prevalence of colistin-resistant *E. coli* strains from the domestic meat samples in our study was lower than the previously reported prevalence of isolates from meat samples in Germany and Brazil (*p* < 0.05) when comparing the prevalence between our study and previous studies by chi-square test [35, 36]. The national sales data for veterinary antibiotics have shown that sales of colistin are relatively low for animal breeding [37]. This may explain why the prevalence of colistin-resistant strains was also low. To the best of our knowledge, this is the first report of such a low prevalence of *mcr-1*-positive *E. coli* isolated from retail meat in Korea. Interestingly, no *mcr-1*-positive *E. coli* isolates have been found from cattle and beef in Korea [16-18,38,39]. In our study, most *mcr-1*-positive *E. coli* were isolated from Brazilian chicken meat samples. In Brazil, chicken meat was previously reported as a reservoir for *mcr-1*-positive *E. coli* [35]. This may explain the high occurrence of *mcr-1*-positive isolates from Brazilian chicken meat. Amongst 51 colistin-resistant isolates, 41 *mcr-1*-negative isolates were identified. The resistance to colistin in these isolates may be associated with chromosomal mutations in PmrAB and PhoPQ, leading to reduce the binding affinity of colistin for its target [6, 7]. However, further studies are needed to elucidate why *mcr-1*-negative isolates presented resistance to colistin.

The *mcr-1* gene in *mcr-1*-positive *E. coli* from domestic pork was located in the IncI2-type plasmid, which was prevalent in Asia [12] and was the type of the first reported *mcr-1*-harboring plasmid (pHNSHP45) from porcine *E. coli* in China [8]. The *mcr-1* genes in *mcr-1*-positive *E. coli* from other sources, such as poultry carcasses, chicken feces, chicken meat, and patients, were reported to be located in IncI2-type plasmids [15, 16, 18, 39]. Furthermore, the *mcr-1*-bearing IncI2 plasmid contig from domestic pork meat was similar to IncI2-type plasmids from other sources, such as livestock, humans, and chicken meat, in Korea [15, 16, 18, 39] (Fig. S1). This suggests that IncI2-type plasmid may play an important role in spreading *mcr-1* gene in Korea.

The *mcr-1* genes in eight *mcr-1*-positive *E. coli* from Brazilian chicken and German pork samples were located in IncX4-type plasmid, which is distributed worldwide [12]. The IncX4 plasmid type was previously found in Brazilian poultry meat samples [35, 40] and in German swine samples [41, 42]. The IncX4-type plasmid was previously detected in *mcr-1*-positive *E. coli* from a diseased swine feces sample in Korea [16, 39].

All but one of *mcr-1* genes located in the IncX4-type plasmids were successfully transferred to the *E. coli* recipient strain. The transfer frequencies of *mcr-1* genes varied, which is comparable with the previous study [39]. A previous study reported that *mcr-1*-bearing, IncI2-type plasmids from *E. coli* were transmissible to other gram-negative bacteria such as *Salmonella* and *Klebsiella* as well as *E. coli* with colistin resistance [18]. However, a previous study showed that the transferability of the *mcr-1*-bearing plasmid depended on the recipient strain or species rather than the plasmid type [43]. The reason for the nontransferability of *mcr-1* gene located in the IncX4 plasmid of EC2016_I115 strain was not determined in our study.

MLST and PFGE are powerful molecular typing techniques for tracking genetic relatedness [44]. In this study, *mcr-1*-positive *E. coli* strains showed very diverse STs and PFGE pulsotypes, presumably indicating that they originated from different clones. ST5229, identified from *E. coli* in domestic pork meat (EC2018_100), was found in porcine *E. coli* in Spain and human *E. coli* in Hong Kong [45, 46]. ST5229 belongs to the clonal MLST complex of ST101, which was previously found in New Delhi metallo-β-lactamase-producing *E. coli* from humans and *mcr-1*-carrying *E. coli* from pig feces in Korea [17, 47]. STs in *mcr-1*-positive *E. coli* from imported meat were previously found from livestock, humans, and food in Asia, Europe, America, and Australia (enterobase.warwick.ac.kr) and were either rare or widespread.

A limitation of this study is the sampling design of colistin-resistant and *mcr-1*-positive *E. coli* strains. Due to the fact that just one *E. coli* strain per meat sample was selected, the prevalence of colistin-resistant and *mcr-1*-positive *E. coli* strains in our study could be underestimated. Despite these limitations, this study provides a comprehensive overview of *mcr-1*-positive *E. coli* diversity and common plasmid-type-bearing *mcr-1* in retail meat in Korea.

In this study, we describe the prevalence and characteristics of *mcr-1*-positive *E. coli* isolated from domestic and imported meat samples in Korea. Our data showed that the prevalence of *mcr-1*-positive *E. coli* strains from retail meat was 0.8%. The *mcr-1*-positive strains from retail meat samples exhibited diverse STs and PFGE pulsotypes, suggesting that the strains had evolved from different *E. coli* clones. However, the *mcr-1* genes in our study were located in the specific types of plasmid (IncI2 and IncX4) and these plasmid types bearing *mcr-1* were also found in *mcr-1*-positive *E. coli* from other sources, including humans, animals, and chicken meat in Korea. These findings suggested that the specific types of plasmid may play an important role in spreading plasmid-mediated colistin resistance in Korea. The mcr-harboring plasmid may contribute to the spread of colistin resistance and the emergence of pandrug-resistant pathogens due to its high transferability to other strains. Thus, retail meat may pose a health risk to consumers and food handlers despite the low prevalence of *mcr-1*-positive *E. coli* from retail meat in Korea, if contaminated with plasmid-mediated colistin-resistant strains. Therefore, close surveillance of *mcr-1*-positive strains should be continued to establish a containment strategy for preventing the spread of colistin resistance throughout the food chain.

## Supplemental Material



Supplementary data for this paper are available on-line only at http://jmb.or.kr.

## Figures and Tables

**Table 1:** Prevalence of colistin-resistant and *mcr-1*-positive *E. coli* isolates from retail meat.

Category	Prevalence of colistin-resistant *E. coli* (no. of resistant isolates/no. of tested isolates)				Prevalence of *mcr-1*-positive *E. coli*, % (no. of positive isolates/no. of tested isolates)			
	
	Domestic	Imported	Total	*P* value^a^	Domestic	Imported	Total	*P* value^a^
Beef	2.6 (9/343)	2.6 (2/78)	2.6 (11/421)	0.9762	0.0 (0/343)	0.0 (0/78)	0.0 (0/421)	> 0.9999
Pork	6.0 (8/134)	5.6 (6/107)	5.8 (14/241)	0.9048	0.7 (1/134)	0.9 (1/107)	0.8 (2/241)	0.8728
Chicken	3.9 (16/414)	7.8 (10/129)	4.8 (26/543)	0.0710	0.0 (0/414)	6.2 (8/129)	1.5 (8/543)	< 0.0001
Total	3.7 (33/891)	5.7 (18/314)	4.2 (51/1205)	0.1247	0.1 (1/891)	2.9 (9/314)	0.8 (10/1205)	< 0.0001

^a^*P* value, difference between the proportions of strains from domestic and imported meat samples by Chi-squared test.

**Table 2:** Antimicrobial resistance of colistin-resistant *E. coli*.

Antibiotics	Source	Range tested (μg/ml)	Break points (μg/ml)	MIC_50_^a^	MIC_90_^a^	Resistance % (n)	*P* value^b^
AmC	*mcr-1*-positive strains (n=10)	2/1-32/16	≥ 32/16	8/4	32/16	20.0 (2)	0.73
	*mcr-1*-negative strains (n=41)			8/4	> 32/16	31.7 (13)	
	subtoal (n=51)			8/4	> 32/16	29.4 (15)	
AMP	*mcr-1*-positive strains (n=10)	2-64	≥ 32	128	> 64	60.0 (6)	0.67
	*mcr-1*-negative strains (n=41)			8	> 64	46.3 (19)	
	subtoal (n=51)			8	> 64	49.0 (25)	
CIP	*mcr-1*-positive strains (n=10)	0.13-16	≥ 4	0.5	8	20.0 (2)	0.63
	*mcr-1*-negative strains (n=41)			≤ 0.13	8	34.1 (14)	
	subtoal (n=51)			≤ 0.13	8	31.4 (16)	
CHL	*mcr-1*-positive strains (n=10)	2-64	≥ 32	8	> 64	30.0 (3)	0.87
	*mcr-1*-negative strains (n=41)			8	> 64	39.0 (16)	
	subtoal (n=51)			8	> 64	37.3 (19)	
COL	*mcr-1*-positive strains (n=10)	2-32	> 2	8	16	100.0 (10)	ND^c^
	*mcr-1*-negative strains (n=41)			32	> 32	100.0 (41)	
	subtoal (n=51)			32	> 32	100.0 (51)	
CTF	*mcr-1*-positive strains (n=10)	0.5-8	≥ 8	≤ 0.5	> 8	20.0 (2)	1.00
	*mcr-1*-negative strains (n=41)			≤ 0.5	8	17.1 (7)	
	subtoal (n=51)			≤ 0.5	> 8	17.6 (9)	
FOX	*mcr-1*-positive strains (n=10)	1-32	≥ 32	8	32	20.0 (2)	1.00
	*mcr-1*-negative strains (n=41)			8	> 32	17.1 (7)	
	subtoal (n=51)			8	> 32	17.6 (9)	
GEN	*mcr-1*-positive strains (n=10)	1-64	≥ 16	≤ 1	16	20.0 (2)	1.00
	*mcr-1*-negative strains (n=41)			2	32	14.6 (6)	
	subtoal (n=51)			2	32	15.7 (8)	
NAL	*mcr-1*-positive strains (n=10)	2-128	≥ 32	8	> 128	30.0 (3)	0.87
	*mcr-1*-negative strains (n=41)			4	> 128	39.0 (16)	
	subtoal (n=51)			4	> 128	37.3 (19)	
STR	*mcr-1*-positive strains (n=10)	16-128	≥ 32	≤ 16	128	50.0 (5)	1.00
	*mcr-1*-negative strains (n=41)			≤ 16	> 128	48.8 (20)	
	subtoal (n=51)			≤ 16	> 128	49.0 (25)	
SXT	*mcr-1*-positive strains (n=10)	0.13/2.4-4/76	≥ 4/76	0.25	> 4/76	30.0 (3)	1.00
	*mcr-1*-negative strains (n=41)			≤ 0.13/2.4	> 4/76	26.8 (11)	
	subtoal (n=51)			≤ 0.13/2.4	> 4/76	26.8 (14)	
TET	*mcr-1*-positive strains (n=10)	2-128	≥ 16	≤ 2	64	40.0 (4)	0.67
	*mcr-1*-negative strains (n=41)			32	128	53.7 (22)	
	subtoal (n=51)			32	128	51.0 (26)	
MDR^c^	*mcr-1*-positive strains (n=10)	ND	ND	ND	ND	60.0 (6)	0.90
	*mcr-1*-negative strains (n=41)	ND	ND	ND	ND	68.3 (28)	
	subtoal (n=51)	ND	ND	ND	ND	66.7 (34)	

^a^MIC_50_ and MIC_90_ are the concentration at which 50% and 90% of the isolates were inhibited.

^b^*P* value, difference between the proportions of *mcr-1*-positive and *mcr-1*-negative strains among colistin-resistant *E. coli* by Chi-squared test.

^c^Abbreviations: MDR, multidrug resistance; ND, not determined.

**Table 3:** Genetic features of *mcr-1*-positive *E. coli* isolated from retail meat.

Strain	Source	Year	Resistance phenotype^a^	Resistance genes	Plasmid replicons^b^	*mcr-1* gene transfer
EC2015_I58	Chicken (Brazil)	2015	COL, STR	*mcr-1.1*, *aadA1*, *mdf(A)*, *qnrB19*	Col(pHAD28), IncFⅠB(K), IncFⅡ(29), **IncX4**	Yes
EC2016_I15	Chicken (Brazil)	2016	AmC, AMP, COL, FOX, NAL, STR, SXT	*mcr-1.1*, *aph(3'')-Ib*, *aph(6)-Id*, *bla*_TEM-1B_, *mdf(A)*, *sul2*, *dfrA14*	IncFⅠB(AP001918), IncFⅡ, **IncX4**	Yes
EC2016_I103	Pork (Germany)	2016	AMP, CHL, COL, TET	*mcr-1.1*, *aadA2b*, *bla*_TEM-1B_, *mdf(A)*, *qnrS1*, *tet(A)*, *dfrA8*	IncR, **IncX4**	Yes
EC2016_I115	Chicken (Brazil)	2016	CIP, COL, GEN, NAL, STR, TET	*mcr-1.1*, *aac(3)-Ⅴia*, *aadA1*, *mdf(A)*, *sul1*, *tet(A)*	IncFⅠB(AP001918), IncFⅡ(SE11), **IncX4**	No
EC2016_I119	Chicken (Brazil)	2016	AmC, AMP, CHL, COL, FOX, STR, SXT	*mcr-1.1*, *aadA1*, *aadA2*, *bla*_TEM-1A_, *Inu(A)*, *mdf(A)*,*cmlA1*, *qnrB19, sul3, dfrA12*	Col(pHAD28), IncFⅠB(AP001918), IncI1-Ⅰ, IncI2, IncX1, **IncX4**, IncY	Yes
EC2016_I183	Chicken (Brazil)	2016	COL	*mcr-1.1*, *mdf(A)*	**IncX4**	Yes
EC2016_I182	Chicken (Brazil)	2016	COL	*mcr-1.5*, *aadA1*, *aadA2b*, *aph(3')-Ia*, *mdf(A)*,*cmlA1*, *sul3*	IncFⅠB(AP001918), IncI1-Ⅰ, IncI2	Yes
EC2017_I300	Chicken (Brazil)	2017	AMP, COL	*mcr-1.1*, *bla*_SHV-12_, *mdf(A)*	IncFⅠB(AP001918), IncI1-I, IncFⅡ(pRSB107), **IncX4**, p0111	Yes
EC2017_I306	Chicken (Brazil)	2017	AMP, COL, CTF, TET	*mcr-1.1*, *bla*_CTX-M-55_, *fosA3*, *mdf(A)*, *tet(A)*	IncFⅡ(pHN7A8), **IncX4**, p0111	Yes
EC2018_100	Pork (Korea)	2018	AMP, CHL, CIP, COL, CTF, GEN, NAL, STR, SXT, TET	*mcr-1.1*, *aac(3)-IId*, *aadA1*, *bla*_CTX-M-15_, *bla*_TEM-1B_, *Inu(F)*, *mdf(A)*,*cmlA1*, *qnrS1*, *sul2*, *sul3*, *tet(A)*, *tet(M)*, *dfrA12*	IncFⅠB(AP001918), **IncI2**, IncX1	Yes

^a^Underlining indicates resistance by transconjugants.

^b^Bolded plasmid name means replicons found in the same contig as the *mcr-1* gene.
