# Supplementary material for: Prevalence and Genetic Characterization of mcr-1-Positive Escherichia coli Isolated from Retail Meats in South Korea
Source: J Microbiol Biotechnol. 2020 Sep 21;30(12):1862–9. doi: 10.4014/jmb.2007.07008 (PMC9728184; doi:10.4014/jmb.2007.07008)
Supplement: Supplementary file 1 [file JMB-30-12-1862-supple.pdf]

J. Microbiol. Biotechnol.
https://doi.org/10.4014/jmb.2007.07008

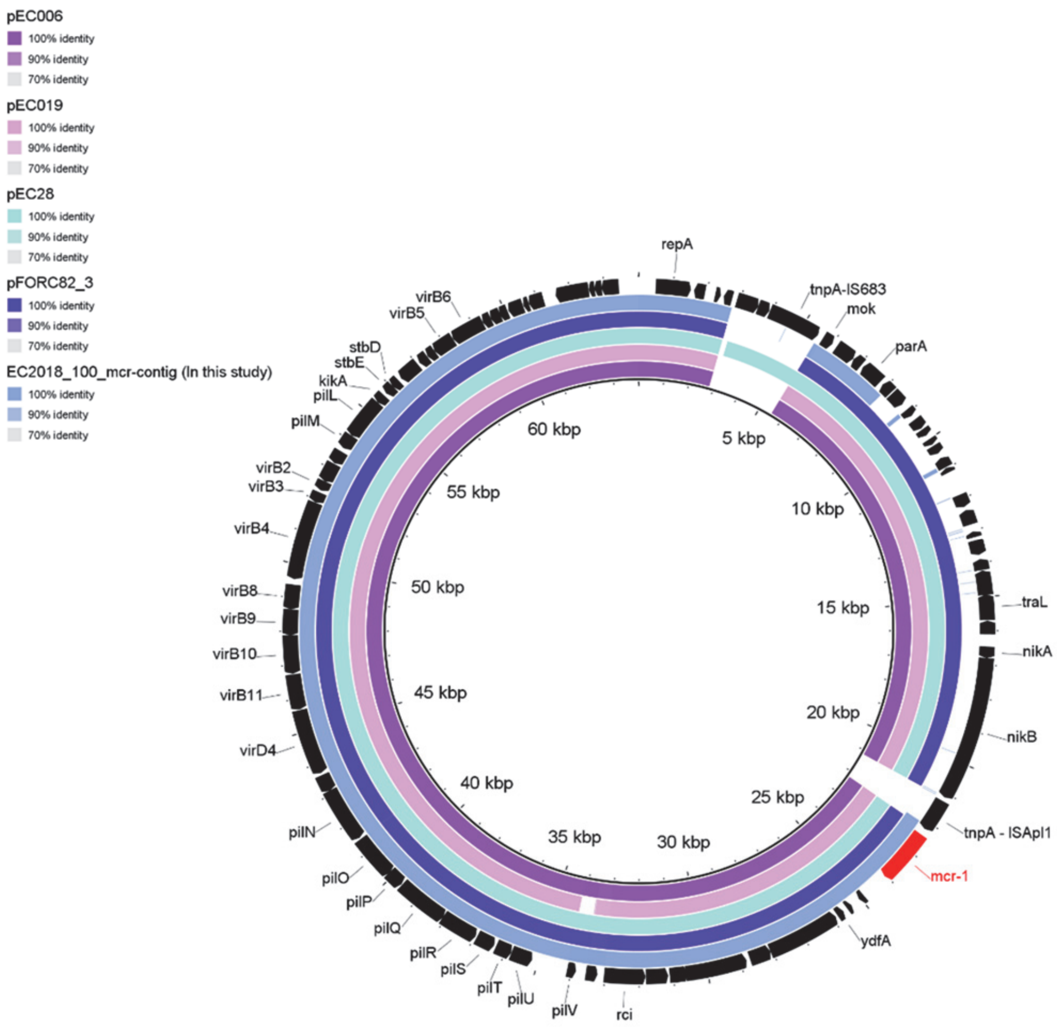

Fig S1. Alignment of the five *mcr-1*-bearing IncI2-type plasmids from *E. coli* strains against reference genome of pHNSHP45 (64 kbp; GenBank accession no. KP347127). The coding regions of the IncI2-type plasmid pHSHP45 from China are represented by alternating red and black arrows in the outermost ring. The concentric rings display similarity between the reference sequence and the other sequences. pEC006, plasmid of *E. coli* from chicken carcass in Korea (96% query coverage, 99.98% identity; 62 kbp; GenBank accession no. KY471144); pEC019, plasmid of *E. coli* from chicken carcass from Korea (95% query coverage, 99.98% identity; 62 kbp; GenBank accession no. KY471145); pEC28, plasmid of *E. coli* from human blood in Korea (96% query coverage, 99.99% identity; 63 kbp; GenBank accession no. KY405001); pFORC82_3, plasmid of *E. coli* from chicken meat in Korea (96% query coverage, 99.99% identity; 65 kbp; GenBank accession no. CP026644); EC2018_100_mcr-contig, *mcr-1*-bearing IncI2 plasmid contig from pork meat in Korea (in our study; 49 kbp).

Table S1. Retail meat samples collected between 2015 and 2018

Category	Province or country[a]	No. of samples		
		Beef	Pork	Chicken
Domestic	Seoul/ Gyeonggi	111	119	104
	Chungcheong	127	126	130
	Gyeongsang	150	140	149
	Jeolla	153	104	102
	Gangwon	71	76	75
	Subtotal	612	565	560
Imported	Australia	407	-	-
	Austria	-	14	-
	Belgium	-	18	-
	Brazil	-	-	232
	Canada	12	48	-
	Chile	-	19	-
	Denmark	-	16	10
	Finland	-	2	-
	France	-	7	-
	German	-	68	-
	Hungary	-	1	-
	Ireland	-	7	-
	Mexico	15	111	-
	Netherland	-	8	-
	New zealand	14	-	-
	Spain	-	158	-
	Thiland	-	-	8
	USA	230	84	8
	Subtotal	678	561	258
Total		1,290	1,126	818

[a] Province and country mean the area for buying domestic meat samples and the exporting country to Korea, respectively.

Table S2. Whole-genome sequences of *mcr-1* -positive *E. coli* from retail meat in Korea

Strain	GenBank accession numbe	Sequencing coverage	Contig no.	Genome size, bp
EC2015_I58	JACABR000000000	62.34×	99	5,176,724
EC2017_I300	JACABY000000000	115.33×	106	5,174,097
EC2016_I182	JACABW000000000	145.65×	83	5,078,329
EC2016_I183	JACABX000000000	142.71×	69	5,082,300
EC2016_I119	JACABV000000000	115.16×	135	5,215,715
EC2016_I103	JACABT000000000	104.56×	124	4,784,941
EC2016_I15	JACABS000000000	124.49×	88	4,899,804
EC2016_I115	JACABU000000000	106.42×	77	4,945,947
EC2017_I306	WVVJ00000000	195.36×	84	4,949,444
EC2018_100	WVVM00000000	178.52×	83	5,084,983

Table S3. MICs and antimicrobial resistance genes (ARGs) of *mcr-1* -positive *E. coli*.

			mcr-1 -positive *E. coli*									
			EC2015_I58	EC2016_I15	EC2016_I103	EC2016_I115	EC2016_I119	EC2016_I182	EC2016_I183	EC2017_I300	EC2017_I306	EC2018_100
Minimum inhibition concentration (MIC), μg/ml	Antimicrobial agents	AmC	8/4	32/16	8/4	4/2	> 32/16	8/4	4/2	8/4	8/4	16/8
		AMP	4	> 64	> 64	2	> 64	4	4	> 64	> 64	> 64
		CIP	1	≤ 0.13	1	8	1	≤ 0.13	≤ 0.13	≤ 0.13	≤ 0.13	> 16
		CHL	8	8	> 64	8	32	16	4	4	8	> 64
		COL	16	8	4	4	> 32	8	8	8	8	8
		CTF	1	1	1	1	1	1	1	1	> 8	> 8
		FOX	16	32	4	4	> 32	4	8	4	8	8
		GEN	1	1	1	16	1	1	1	1	1	> 64
		NAL	16	64	8	> 128	16	2	2	2	2	> 128
		STR	64	128	16	32	128	16	8	16	16	> 128
		SXT	≤ 0.13/2.4	> 4/76	≤ 0.13/2.4	≤ 0.13/2.4	> 4/76	≤ 0.13/2.4	≤ 0.13/2.4	≤ 0.13/2.4	≤ 0.13/2.4	> 4/76
		TET	2	2	64	64	2	2	2	2	64	128
Antimicrobial resistance genes	Aminoglycoside	*aac(3)-IId*										
		aac(3)-Via										
		aadA1										
		aadA2										
		aadA2b										
		aph(3')-Ia										
		aph(3'')-Ib										
		aph(6)-Id										
	Beta-lactam	$bla_{CTX-M-15}$										
		$bla_{CTX-M-55}$										
		bla_{SHV-12}										
		bla_{TEM-1A}										
		bla_{TEM-1B}										
	Colistin	*mcr-1.1*										
		mcr-1.5										
	Macrolide	*lnu(A)*										
		lnu(F)										
		mdf(A)										
	Fosfomycin	*fosA3*										
	Phenicol	*cmlA1*										
	Quinolone	*qnrS1*										
		qnrB19										
	Sulphonamide	*sul1*										
		sul2										
		sul3										
	Tetracycline	*tet(A)*										
		tet(M)										
	Trimethoprim	*dfrA8*										
		dfrA12										
		dfrA14										
Mutations		*gyrA* (D87N)										
		gyrA (S83L)										
		parC (S80I)										

[a] The minimum inhibition concentration (MIC) values with gray background indicate the resistance to the antimicrobial agents. Antimicrobial resistance genes (ARGs) and mutations with blue background indicate the presence of ARGs and mutations.

[b] Mutations refer to nonsynonymous mutations conferring fluoroquinolone or quinolone resistance.
